# RgIA4 Potently Blocks Mouse α9α10 nAChRs and Provides Long Lasting Protection against Oxaliplatin-Induced Cold Allodynia

**DOI:** 10.3389/fncel.2017.00219

**Published:** 2017-07-21

**Authors:** Sean B. Christensen, Arik J. Hone, Isabelle Roux, Julie Kniazeff, Jean-Philippe Pin, Grégory Upert, Denis Servent, Elisabeth Glowatzki, J. Michael McIntosh

**Affiliations:** ^1^Department of Biology, University of Utah Salt Lake City, UT, United States; ^2^Department of Otolaryngology, Head and Neck Surgery, The Center for Hearing and Balance and the Center for Sensory Biology, The Johns Hopkins University School of Medicine Baltimore, MD, United States; ^3^IGF, Centre National de la Recherche Scientifique, Institut National de la Santé et de la Recherche Médicale, Université Montpellier Montpellier, France; ^4^Service d'Ingénierie Moléculaire des Protéines, CEA, Université Paris-Saclay Gif-sur-Yvette, France; ^5^Department of Neuroscience, The Johns Hopkins University School of Medicine Baltimore, MD, United States; ^6^George E. Whalen Veterans Affairs Medical Center Salt Lake City, UT, United States; ^7^Department of Psychiatry, University of Utah Salt Lake City, UT, United States

**Keywords:** nicotinic, chemotherapy, neuropathic pain, α9α10, conotoxins

## Abstract

Transcripts for α9 and α10 nicotinic acetylcholine receptor (nAChR) subunits are found in diverse tissues. The function of α9α10 nAChRs is best known in mechanosensory cochlear hair cells, but elsewhere their roles are less well-understood. α9α10 nAChRs have been implicated as analgesic targets and α-conotoxins that block α9α10 nAChRs produce analgesia. However, some of these peptides show large potency differences between species. Additionally several studies have indicated that these conotoxins may also activate GABA_B_ receptors (GABA_B_Rs). To further address these issues, we cloned the cDNAs of mouse α9 and α10 nAChR subunits. When heterologously expressed in *Xenopus* oocytes, the resulting α9α10 nAChRs had the expected pharmacology of being activated by acetylcholine and choline but not by nicotine. A conotoxin analog, RgIA4, potently, and selectively blocked mouse α9α10 nAChRs with low nanomolar affinity indicating that RgIA4 may be effectively used to study murine α9α10 nAChR function. Previous reports indicated that RgIA4 attenuates chemotherapy-induced cold allodynia. Here we demonstrate that RgIA4 analgesic effects following oxaliplatin treatment are sustained for 21 days after last RgIA4 administration indicating that RgIA4 may provide enduring protection against nerve damage. RgIA4 lacks activity at GABA_B_ receptors; a bioluminescence resonance energy transfer assay was used to demonstrate that two other analgesic α-conotoxins, Vc1.1 and AuIB, also do not activate GABA_B_Rs expressed in HEK cells. Together these findings further support the targeting of α9α10 nAChRs in the treatment of pain.

## Introduction

Neuropathic pain, caused by lesion or disease of the somatosensory system, is estimated to afflict 7–10% of the population (van Hecke et al., 2014). Common causes include traumatic nerve injury, metabolic disorders such as diabetes, viral infection, including herpes or HIV, and nerve injury induced by cancer chemotherapy. Although a variety of treatment options are available, a significant fraction of patients are treatment refractory leaving millions to endure long lasting pain states (Costigan et al., 2009; Colloca et al., 2017). In addition, polypharmacy to control pain can lead to significant side effect burden (Colloca et al., 2017).

Opioids, inspired by opium isolated from the poppy *Papaver somniferum*, are one of the most powerful available pain-reducing medications. Unfortunately, chronic neuropathic pain is not well-treated by opioids. In addition, opioid use is plagued by effect tolerance, addiction, and unintentional overdose. Over 12 million people in the U.S. are estimated to have abused opioids (Brady et al., 2016). Thus, non-opioid alternatives are a high priority.

Classical neuronal nicotinic acetylcholine receptors are ligand-gated ion channels assembled in pentamers composed of various α or α/β subunit combinations. Agonists of nAChRs have been intensively investigated as potential analgesics. The tobacco plant toxin nicotine is a nAChR agonist that has long been known to have antinociceptive properties; however dosage requirements and side effects from non-selective activity on multiple nAChR subtypes preclude practical use as an analgesic. Considerable effort has been expended to develop selective agonists of CNS α4β2 nAChRs and more recently α7 nAChR agonists and positive allosteric modulators as potential therapeutic agents (Umana et al., 2013).

The α9α10 subtype of nAChR has also been proposed as a novel analgesic target (Vincler et al., 2006; Vincler and McIntosh, 2007; McIntosh et al., 2009; Del Bufalo et al., 2014; Mohammadi and Christie, 2014). Interestingly, rather than CNS targeted agonists, peripherally acting α9α10 antagonists have shown promise (Vincler et al., 2006; Holtman et al., 2011; Zheng et al., 2011; Wala et al., 2012; Luo et al., 2015).

A particularly rich source of nAChR antagonist are marine mollusks of the genus *Conus* (Azam and McIntosh, 2009; Lebbe et al., 2014; Dutertre et al., 2017). Cone snails are carnivores that immobilize their prey by injecting them with a complex mixture of toxins. There are ~700 species of *Conus*. Of the species examined thus far, the great majority have documented components that are structurally similar to peptides known to block nAChRs. Thus, *Conus* may have thousands of novel nAChR targeted peptides. A very valuable online database, ConoServer, has documented reported *Conus* sequences (Kaas et al., 2012).

Three *Conus* peptides, that act on α9α10 nAChRs, Vc1.1, RgIA, and GeXIVA and analogs thereof have shown analgesic activity in several models of neuropathic pain (Satkunanathan et al., 2005; Vincler et al., 2006; Clark et al., 2010; Carstens et al., 2011; Di Cesare Mannelli et al., 2014; Luo et al., 2015; Castro et al., 2016; Pacini et al., 2016; Romero et al., 2017). Vc1.1 reached phase II human clinical trials, but was withdrawn in part, because it was discovered that Vc1.1 was much less potent at the human vs. rat α9α10 nAChR (Metabolic, 2007). Thus, species analysis is particularly important for this subgroup of α-conotoxins.

To our knowledge there are no previous reports of the properties of heterologously expressed mouse α9α10 nAChRs. In this report, nAChR subunits α9 and α10 were cloned and we examined the subtype selectivity of a newly developed antagonist α-conotoxin RgIA4 for mouse nAChRs. Further, we demonstrate the long acting analgesic effects of this peptide in a mouse model of chemotherapy induced neuropathy.

## Materials and methods

### Animals

Animals for RNA extraction: 4 day old C57BL/6J mice (stock number 000664, Jackson Laboratory, Bar Harbor, ME, USA) were euthanized, and their inner ears were quickly removed from the temporal bones. All experimental procedures involving animals were approved by the Johns Hopkins University Animal Care and Use Committee. Animals for oxaliplatin experiments: Wild-type mice were CBA/CaJ (Jackson Labs, Bar Harbor ME). Germline α9 knockout (KO) mice (Abazeed et al., 2013) originally on a 129Sv/Ev and CBA/CaJ background were crossed using an accelerated backcrossing program (Jackson Labs) until 99.5% identity with wild-type CBA/CaJ mice was achieved. Mice were then further backcrossed with wild-type CBA/CaJ mice an additional three generations. Experiments involving animals have been reported according to ARRIVE guidelines (Kilkenny et al., 2010). All efforts were made to minimize animal suffering and to reduce the number of animals used. All experimental procedures were in accordance with the National Institutes of Health guidelines for the care and use of laboratory animals and were performed under approved protocols at the University of Utah.

### RNA extractions and reverse transcription-polymerase chain reactions (RT-PCR)

Mouse cochlear neuroepithelia were isolated in RNAlater stabilization solution (ThermoFisher Scientific, Waltham, MA, USA) and quickly frozen. Total RNA was extracted using Trizol reagent (Invitrogen, ThermoFisher Scientific). Reverse transcriptase reactions were performed using Superscript III (ThermoFisher Scientific) with random primers p(dN)_6_ (Roche, Sigma-Aldrich, St Louis, MO, USA) and polymerase chain reaction (PCR) using the high fidelity TaKaRa LA Taq DNA Polymerase (TaKaRa Bio, Mountain View, CA, USA).

Primers 5′-cgACTAGTgttgggaaaggATGaaccggccccatccc-3′ and 5′-cgCTCGAGctaatctgctcttgctatgatcaagacgg-3′ were used to amplify *Chrna9* coding sequence, and primers 5′-cgACTAGTccagcagggcctgttgctttacatctcc-3′ and 5′-cgCTCGAGttacagggcttgcaccagtaccaggaggc-3′, *Chrna10* coding sequence. In both cases, these primers were designed to add SpeI and XhoI restriction sites 5′ and 3′ respectively of the coding sequences and to amplify part of the 5′UTR.

The 1,467 and 1,423 bp fragments amplified were isolated and purified after agarose gel separation and inserted into pGEMT vector (pGEMT Vector System, Promega). Sequences of the clones obtained were compared to the direct sequences of pooled independent PCR amplifications using cochlear neuroepithelia RT-PCR as template, to reference sequences (NM_001081104, NM_001081424), and to C57BL/6J genomic sequences using BLAT—UCSC Genome Browser (University of California—Santa Cruz, CA, USA) (GRCm38/mm10).

**Figure 1:**
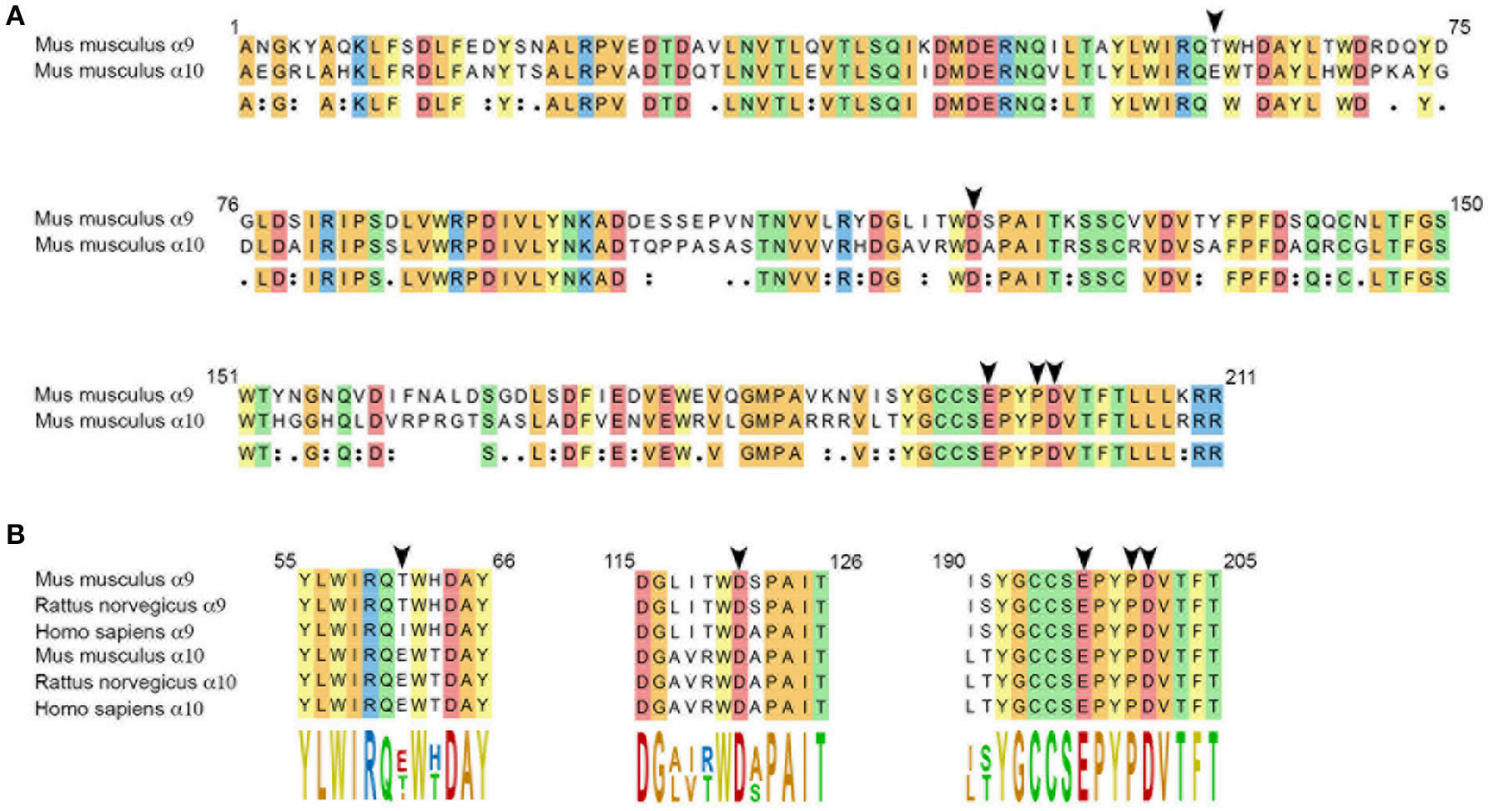
**(A)** Amino acid sequence alignment of the extracellular domains of mouse α9 and α10 subunits. Numbering complies with the numbering of the α9 nAChR subunit presented in the crystal structure of the human α9 extracellular domain (Zouridakis et al., 2014). Arrow heads point at residues thought to interact with α-CT× RgIA4: T61 and D121 (α9) and E197, P200, and D201 (α10), based on comparison with the binding of rat α9 and α10 subunits to α-conotoxin RgIA (Perez et al., 2009; Azam et al., 2015). Conserved residues are highlighted, colors were chosen depending on the chemical properties of the residues. **(B)** Sequence alignments of the α-conotoxin RgIA–interacting domains of mouse, rat and human α9, and α10 nAChR subunits.

The sequence alignments presented in Figure 1 were obtained using Clustal Omega (Sievers et al., 2011) (MegAlign Pro, DNASTAR Lasergene, Madison, WI, USA). The following sequences were used as reference: *Mus musculus* (NP_001074573, NP_001074893), *Rattus norvegicus* (NP_075219, NP_072161), *Homo sapiens* (NP_060051, NP_065135) for α9 and α10 subunits, respectively.

### Plasmid constructs and preparation

*Chrna9* and *Chrna10* cDNAs were further subcloned into a modified pSGEM vector (originally developed by Dr. Michael Hollmann, Ruhr University, Bochum, Germany), using SpeI and XhoI restriction sites. This vector allows the expression of a transcript including the 5'UTR of the β-globin of *Xenopus laevis*, the coding sequence of the nAChR subunit preceded by its native 5'UTR, and followed by the 3'UTR of the β-globin of *X. laevis*. It was optimized to increase the expression (translational efficiency) of the nAChR subunit in oocytes (Filchakova and McIntosh, 2013).

Plasmid DNA were isolated from bacteria using Maxiprep Qiagen plasmid kits (Qiagen, Hilden, Germany). The sequences of the expression plasmids were confirmed by automated DNA sequencing in the Johns Hopkins University School of Medicine DNA sequencing Core facility.

### Agonist concentration response analysis

*X. laevis* oocytes were injected with 25 ng of each cRNA encoding α9 and α10 subunits as previously described (Cartier et al., 1996) and incubated for 48 h at 17°C in ND96 prior to use. Two-electrode voltage clamp electrophysiology was conducted to assess for functional expression of α9α10 nAChRs as previously described (Azam et al., 2008). The oocyte membranes were clamped at a holding potential of −70 mV and stimulated with 1 s pulses of 1 mM ACh once every 60 s. After a steady-state response baseline was observed, the oocytes were then stimulated with ascending concentrations of ACh, choline, or nicotine. Each oocyte was stimulated first with ACh followed by choline then nicotine. The maximal response value for activation by ACh was determined using the Hill equation: Y = Bottom + (Top-Bottom)/(1+10^∧^((LogEC_50_−X)*Hill slope)). The responses to all concentrations of ACh, choline, and nicotine were then normalized to this value and calculated as a % response.

### Antagonist response analysis

*Xenopus laevis* oocytes were injected with 15–30 ng of cRNA (equal amounts for each nAChR subunit) and incubated for 48 h at 17°C in ND96 prior to use. The mouse α7 subunit was co-injected with an equal amount of ric3 to increase expression. The mouse α9 and α10 subunits were made as a part of this study;all other mouse clones were kindly provided by Dr. Jerry Stitzel (University of Colorado, Boulder, CO, USA). Two-electrode voltage clamp electrophysiology was used with oocyte membranes clamped at a holding potential of –70 mV. Oocytes were stimulated with 1 s pulses of ACh every 60 s at the following concentrations: for adult and fetal muscle 10 μM was used; for mouse α7, 200 μM; and for all other subunit combinations 100 μM ACh was used. After a steady baseline of ACh pulses was achieved using ND96, the solution was switched to ND96 containing various toxin concentrations and ACh pulses were observed for toxin response. All dose response curves, including IC_50_ and hillslope data, were calculated using non-linear regression (curve fit) sigmoidal dose response (variable slope), (GraphPad Prism, San Diego, CA, USA).

### Oxaliplatin-induced cold allodynia

Oxaliplatin (MedChem Express, Monmouth Junction, NJ) was dissolved at 0.6 μg/μl in 0.9% NaCl. RgIA4 was dissolved at 0.01 μg/μl in 0.9% NaCl. Mice were injected i.p. daily with oxaliplatin (3.5 mg/kg) or 0.9% saline (vehicle). Additionally, mice were injected s.c. with RgIA4 (40 μg/kg) or vehicle. Treatment weeks included injection on Thurs, Fri, and Mon–Wed. On Thurs, 24 h after last injection, mice were assessed for cold allodynia. Injections stopped on day 21, with assessment occurring on day 22 and subsequent testing occurring once a week for three additional weeks. Testing was conducted using a cold plate test chamber (IITC, Inc Life Science, Woodland, CA). Animals were allowed to acclimate in the chamber at room temperature (23°C) for 5 min. Temperature was then lowered at a rate of 10°C per minute. The testing was stopped when the animal lifted both forepaws and shaking or licking occurred. Alternating lifting of forepaws was not scored. Throughout the study period, experimenters were blinded as to the identity of the injected compounds.

### Competition binding assay

A competition binding assay on intact HEK293 cells was performed using the Tag-lite™ technology (Cisbio bioassays) (Zwier et al., 2010). HEK293 cells were transfected with plasmids encoding the rat GABA_B1_ subunit fused to a Snap tag at the N-terminus and the rat GABA_B2_ subunit and seeded in 96-well black-walled plates at a density of 100,000 cells per well. Twenty-four hours after transfection, the cells were labeled with 300 nM SNAP-Lumi4Tb (Cisbio bioassays, Codolet, France) for 1 h at 37°C in Tag-lite buffer. After extensive washing with Tag-lite buffer, the cells were incubated with the indicated α-conotoxins. Non-fluorescent ligands (GABA or CGP54626) or buffer together with 10 nM fluorescent CGP54626-Red (Cisbio bioassays, Codolet, France) were incubated for 3 h at 4°C prior to signal detection. The fluorescence was collected at 620 and 665 nm using a Pherastar plate reader (BMG Labtech, Ortenberg, Germany), 50 ms after laser excitation at 337 nm. The FRET signal was then calculated as the ratio (signal at 665 nm)/(signal at 620 nm) × 10^4^ and normalized to specific binding. Non-specific binding was determined in the presence of a high concentration of unlabeled CGP54626 (1 μM).

### Bioluminescence resonance energy transfer assay

Bioluminescence Resonance Energy Transfer (BRET) assay was performed on HEK293 cells by recording heterotrimeric G protein dissociation following receptor activation as previously described (Gales et al., 2006). Briefly, HEK293 cells were transfected with plasmids encoding the rat receptor subunits (GABA_B1a_ and GABA_B2_) and the G protein heterotrimer fused to BRET chromophores [Gα_i1_-Rluc or Gα_oA−_Rluc, Gβ2 and Gγ2-Venus (Comps-Agrar et al., 2011)]. Cells were seeded in a 96-well white plates at a density of 100,000 cells per well. Twenty-four hours after transfection, the cells were washed with phosphate buffered saline prior to Coelenterazine *h* substrate (5 μM) and GABA (1 mM) or α-conotoxin (1 μM) addition. The BRET signal was recorded over time using the Mithras LB 940 plate reader (Berthold Biotechnologies, Bad Wildbad, Germany) that allows the sequential integration of light signals detected with two filter settings (Rluc filter, 485 ± 20 nm and Venus/YPF filter, 530 ± 25 nm). The BRET signal is determined as the ratio between Venus and Rluc emissions.

## Results

### Heterologously expressed mouse α9 and α10 subunits form functional nAChRs

In the developing mouse cochlea, an ACh response and cholinergic efferent activity with pharmacology consistent with activation of α9 and α10 nAChR subunits, can be detected in cochlear inner hair cells at P4 (Roux et al., 2016). We cloned the cDNAs of α9 and α10 subunits expressed in P4 C57BL/6J mouse cochlear neuroepithelium (see *Materials and Methods*). At the protein level, α9 isolated from mouse showed 97% identity with rat, and 91% identity with human homologs. α10 showed 98% identity with rat and 92% identity with human homologs. Sequences of the extracellular domains of mouse α9 and α10 are shown in Figure 1. Residues previously determined to be critical for α-conotoxin RgIA binding (Azam et al., 2015) are indicated by arrowheads in Figure 1B. Note the complete conservation of these residues between rat and mouse sequences.

**Figure 2:**
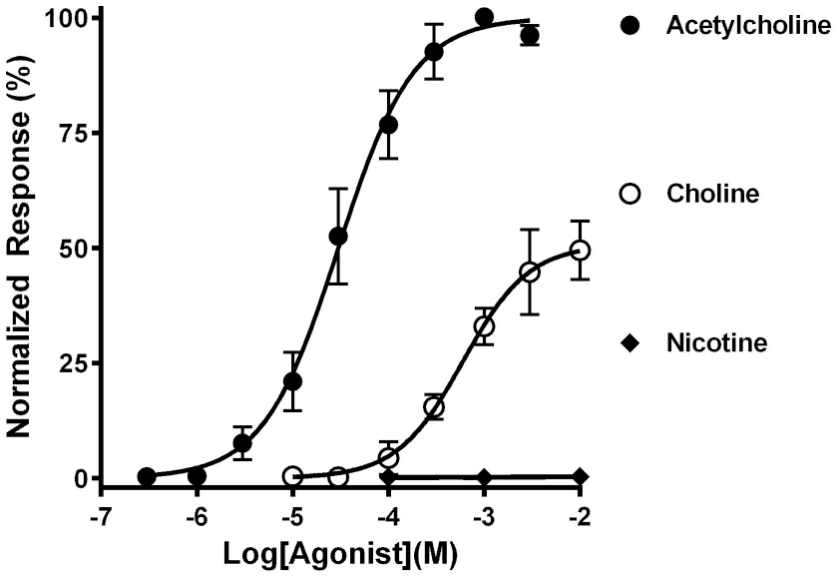
Functional characterization of mouse α9α10 nAChRs heterologously expressed in *Xenopus laevis* oocytes. Oocytes expressing mouse α9α10 nAChRs were subjected to two-electrode voltage clamp electrophysiology as described in *Materials and Methods*. The EC_50_for activation of α9α10 nAChRs by ACh was 30.1 (23.5–38.7) μM and the Hill slope was 1.1 (0.8–1.4) (*n* = 5). The EC_50_ for activation by choline was 601 (311–1160) μM; Hill slope 1.3 (0.5–2.1) maximal efficacy was 50 ± 5% relative to ACh (*n* = 4). Nicotine failed to evoke currents in all oocytes tested (0.4 ± 0.2%) (*n* = 4). The values in parenthesis denote the 95% confidence interval.

Each cDNA insert was subcloned into the pSGEM *Xenopus* oocyte expression vector and used for cRNA preparation. The cRNAs were then injected into oocytes to assess whether mouse α9 and α10 subunits could assemble into functional receptors. The oocytes were subjected to two-electrode voltage clamp electrophysiology and assessed for functional responses to nicotinic agonists (Figure 2). Under these conditions, the oocytes responded robustly to 1 mM ACh as evidenced by current amplitudes that were often in excess of 10 μA. Choline also evoked currents in these oocytes and behaved as a partial agonist relative to ACh. Nic on the other hand failed to evoke currents at concentration from 100 nM to 10 mM. This pharmacological profile is similar to that reported for heterologously expressed human and rat α9-containing nAChRs (Elgoyhen et al., 1994, 2001; Verbitsky et al., 2000; Sgard et al., 2002).

### RgIA4 potently and selectively blocks mouse α9α10 nAChRs

**Figure 3:**
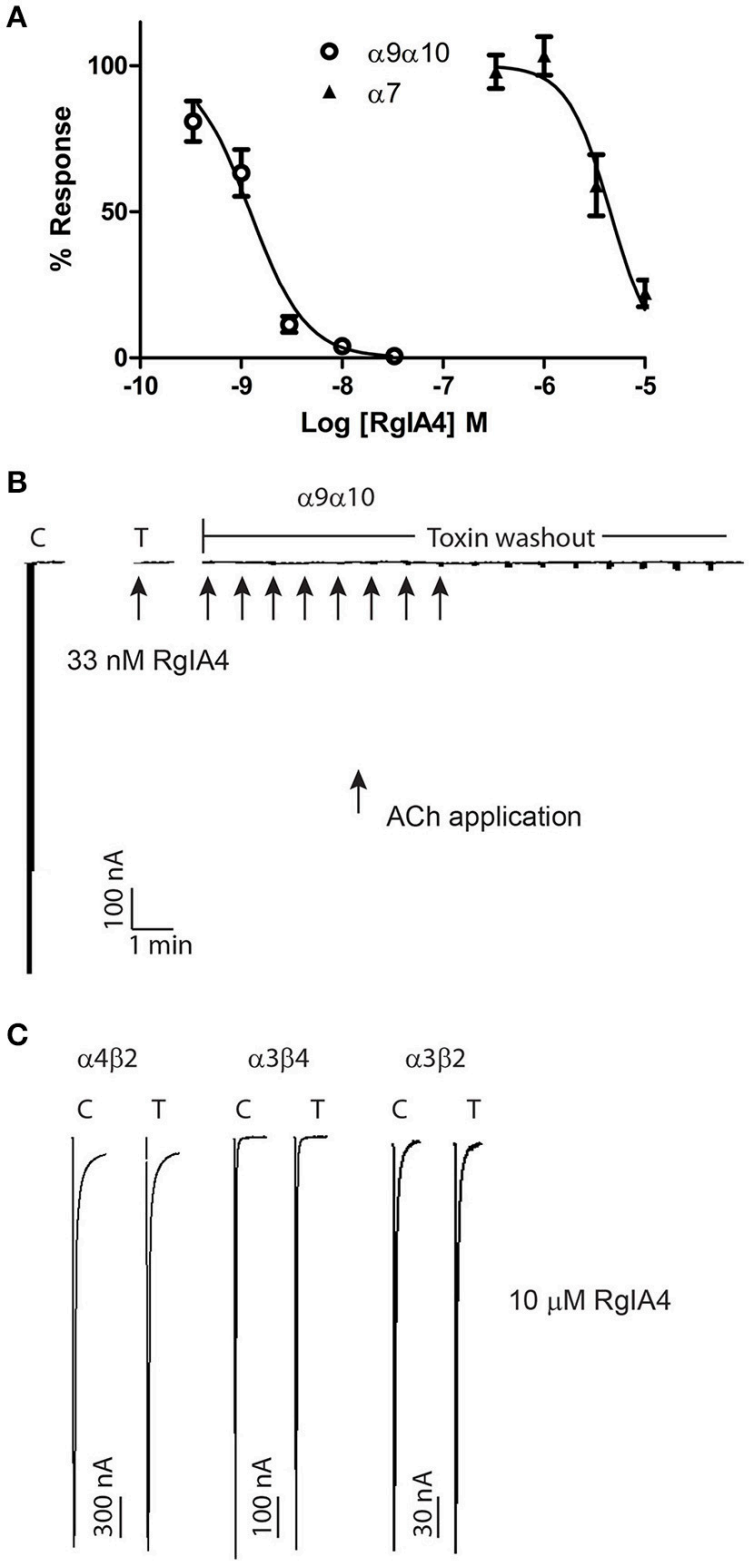
RgIA4 selectively blocks mouse α9α10 nAChRs. nAChRs were expressed in *Xenopus laevis* oocytes. **(A)** The concentration response curves for α9α10 and α7 are shown. The IC_50_ for α9α10 was 1.2 (0.95–1.6) nM with a Hill slope of 1.6 (0.91–2.2). The IC_50_ for α7 was 4.5 (3.1–6.4) μM with a Hill slope of 2.0 (0.84–3.2). The values in parenthesis denote the 95% confidence intervals; *n* = 3–7 for each condition. **(B)** Recovery from toxin block of α9α10 is relatively slow. Example trace of block by 33 nM RgIA4. Recovery after 15 min toxin washout was ~2%. Arrows indicate application of 10 μM ACh. **(C)** At all other indicated nAChR subtypes, RgIA4 (10 μM) blocked less than 50% of the ACh-evoked response; the percent of the ACh-response for each subtype was α1β1δϵ, 95.0 ± 3.8; α1β1δγ, 86.2 ± 3.8; α3β2, 106 ± 2.9; α3β4, 105 ± 0.7; α4β2, 102 ± 3.3; α4β4, 74.3 ± 3.0. *n* = 3–4 for each condition; ± is S.E.M. Example traces for α4β2, α3β4, and α3β2 are shown. Results are summarized in Table 1.

α-conotoxins that block the α9α10 nAChR were previously shown to vary widely in their potency for rat vs. human nAChRs, with these peptides being orders of magnitude less potent on the human α9α10 nAChR. We therefore assessed the potency of RgIA4 on mouse α9α10 nAChRs. RgIA4 potently blocked the ACh response of the α9α10 nAChR subtype with an IC_50_ of 1.2 nM (Figure 3). α-conotoxin [V11L;V16D]ArIB is a potent antagonist of homomeric α7 nAChRs (Whiteaker et al., 2007). Other antagonists of α7 nAChRs, including methyllycaconitine (MLA) and α-bungartoxin also potently block α9α10 nAChRs. We therefore assessed the activity of α-conotoxin [V11L;V16D]ArIB on mouse α9α10 nAChRs. In contrast to RgIA4, [V11L;V16D]ArIB (10 μM) blocked only 12 ± 8.9% of 100 μM ACh-induced current. Thus, RgIA4 is > 10,000-fold more potent than α-conotoxin [V11L;V16D]ArIB on mouse α9α10 nAChRs.

The activity of RgIA4 was further assessed on additional mouse nAChR subtypes including the fetal and adult muscle subtypes α1β1δγ, α1β1δϵ, and neuronal subtypes α2β2, α3β2, α3β4, α2β4, α4β2, α4β4, and α7 as described in *Materials and Methods*. For each of the α/β subunit heteromers, 10 μM peptide blocked less than 50% of current (Table 1). The IC_50_ for α7 was 4.5 μM (95% confidence interval 3.1–6.4 μM). Block of the α9α10 nAChR by a low concentration (33 nM) RgIA4 abolished ACh-induced current. Recovery from block following peptide washout was very slow (~2% at 15 min, Figure 3). In contrast, recovery from block of α7 was rapid. After two min of washout of 5 μM RgIA4 (the approximate IC_50_ concentration), the ACh current recovered to 101.6 ± 6.9% of pre-compound application baseline (*n* = 3, data not shown).

**Table 1:** RgIA4 IC_50_ values for various nAChR subtypes.

**nAChR Subtype**	**IC_50_ (nM)**
α1β1δϵ	>10,000
α1β1δγ	>10,000
α2β2	>10,000
α3β2	>10,000
α3β4	>10,000
α4β2	>10,000
α4β4	>10,000
α7	4,500
α9α10	1.2

*Xenopus laevis oocytes were injected with nAChR subunits to express the indicated subtypes. RgIA4 was then applied to the oocytes expressing the nAChR subtypes and the response to ACh was assessed*.

### RgIA4 provides long lasting protection against chemotherapy induced cold allodynia

**Figure 4:**
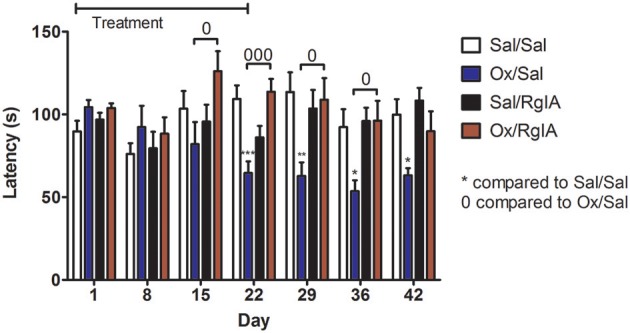
RgIA4 prevents oxaliplatin-induced cold allodynia. Mice were injected once per day with oxaliplatin (3.5 mg/kg i.p) or 0.9% saline (vehicle) for control animals. In addition, mice were injected once per day with RgIA4 (40 μg/kg, s.c.) or vehicle. During the treatment period, mice were injected for 2 days, followed by a 2 day break, followed by injection for another 3 days. Twenty-four hours after last injection, mice were assessed for cold allodynia using a cold-plate as described in *Materials and Methods*. This injection schedule was repeated for two additional weeks. Treatments were stopped on day 21; cold-plate assessment was performed 24 h later on day 22; subsequent testings were performed once a week for three additional weeks. Time to respond to decreasing temperature was measured and indicated on the y axis as the mean ± SEM (*n* = 8) for each experimental group. Experimenters were blinded to treatment conditions. Data was analyzed using a one-way ANOVA with Dunnett's Multiple Comparison Test, *P*-values were indicated as: ^*^P < 0.05, ^**^P < 0.01, and ^***^P < 0.001 for significant difference from vehicle/vehicle control. And ^0^*P* < 0.05, ^000^*P* < 0.001, and indicating significant difference compared with oxaliplatin/vehicle mice.

Chemotherapy induced neuropathy is a common and dose limiting side effect of cancer treatment. Pain from relatively mild cold temperatures (cold allodynia) is particularly problematic following oxaliplatin treatment. There are no FDA approved medications to prevent this complication. RgIA4 was previously shown to prevent cold allodynia in oxaliplatin treated rats and mice. Effects lasted up to 72 h post-treatment (Romero et al., 2017). An unanswered question is whether the RgIA4 prevention of cold allodynia represented temporary delay in development of the oxaliplatin-induced side effect or was indicative of more long lasting effects. Here we evaluated the effects of 3 weeks of treatment with RgIA4 followed by a further 3 weeks of observation. Four groups of mice were treated with either saline + saline, oxaliplatin + saline, saline + RgIA4 or oxaliplatin + RgIA4. This treatment regimen continued for 3 weeks after which all treatments were stopped. Mice were assessed at weekly intervals during and after treatment for the presence of cold allodynia. Experimenters were blinded to treatment conditions. Oxaliplatin produced cold allodynia, an effect that significantly differed from control beginning by week three and continuing for 2 weeks post-oxaliplatin treatment. Oxaliplatin/RgIA4 treated animal differed significantly from oxaliplatin/saline treated animals as measured at weeks 2–5. RgIA4 treated animals did not differ from saline treated controls at any tested time point (Figure 4).

### Binding and functional activity of structurally related conotoxins on GABA_B_ receptors

**Figure 5:**
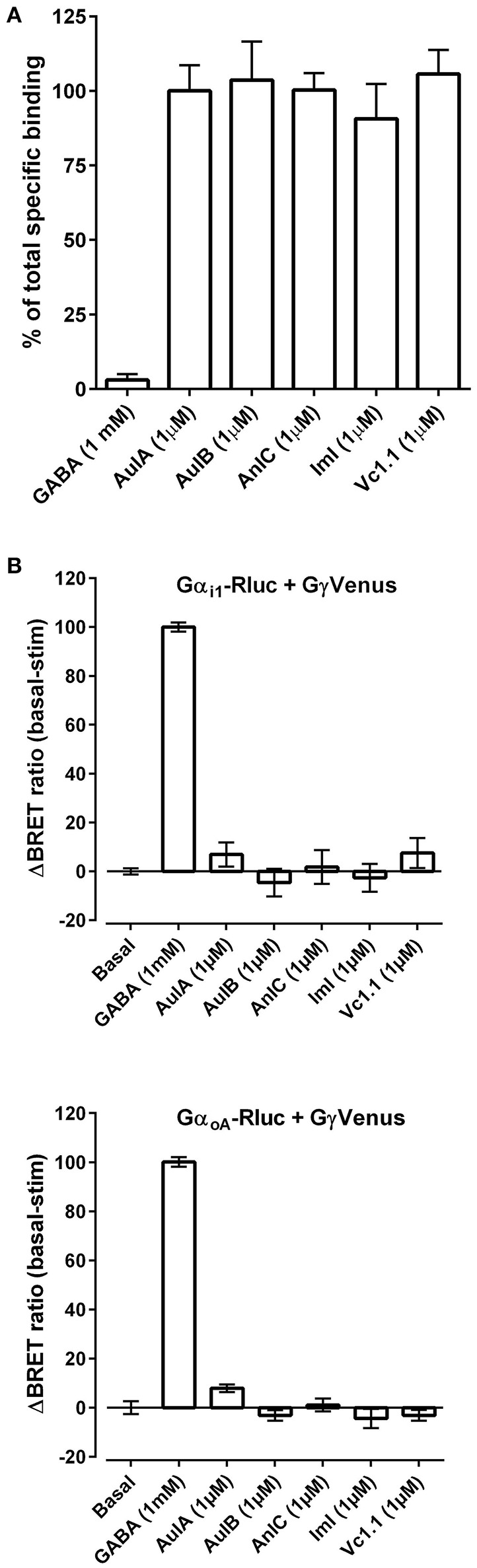
**(A)** α-Conotoxins do not displace the orthosteric GABA_B_R antagonist CGP54626-Red. GABA, CGP54626, and the indicated α-Ctxs (at 1 μM) were assessed for the ability to displace CGP54626-Red binding from GABA_B_R heterologously expressed in HEK293 cells as described in *Material and Methods*. Three replicates were obtained for each value and the experiment was repeated three times. **(B)** α-Conotoxins do not activate GABA_B_R. The indicated α-conotoxins were tested at 1 μM for their ability to activate GABA_B_R heterolgously expressed together with fluorescently tagged G protein subunits in HEK293 cells. At resting state, the G-protein subunits are in close proximity resulting in a strong basal bioluminescence resonance energy transfer (BRET) signal. Activation of GABA_B_R was assessed by monitoring dissociation of Gα_i1_-Rluc (top) or Gα_oA_-Rluc (bottom) from β2γ2-Venus as measured by the change in BRET. The results after 10 min of toxin incubation are expressed as a percentage of GABA-induced BRET change from basal fluorescence levels. Three replicates were obtained for each value and the experiment was repeated three times.

RgIA4 was chosen for the present study because it potently binds α9α10 nAChRs but not multiple other tested targets including opioid receptors or GABA_B_ receptors (GABA_B_R) (Romero et al., 2017). In addition to RgIA4, other structurally related nAChR targeted α-conotoxins are analgesic in several models of neuropathic pain. Among these α-conotoxins, the α9α10 antagonist Vc1.1 has been repeatedly shown to be analgesic. Several studies have shown that Vc1.1 blocks N-type calcium channels in dorsal root ganglion, an effect blocked by GABA_B_ antagonists. In addition, GABA_B_ antagonists prevented analgesic effects of Vc1.1, strongly implicating GABA_B_ receptors as contributing to the therapeutic effects (Callaghan et al., 2008; Callaghan and Adams, 2010; Klimis et al., 2011; Huynh et al., 2015; Castro et al., 2016). However, other studies have failed to confirm GABA_*B*_ agonist activity (McIntosh et al., 2009; Napier et al., 2012; Wright et al., 2015), prompting debate as to whether the analgesic actions of Vc1.1 and related peptides occur via block of α9α10 nAChRs or stimulation of GABA_B_R (Adams et al., 2012; Mohammadi and Christie, 2015). Separately, AuIB was shown to be analgesic, to stimulate GABA_B_Rs but not to block α9α10 nAChRs (Klimis et al., 2011). To further examine the structure, function relationship of conotoxins acting on GABA_B_Rs we first conducted a fluorescent ligand competition binding assay using GABA_B_Rs expressed in HEK293 cells. Lumi4-Tb-labeled GABA_B_Rs were incubated with the fluorescent ligand CGP54626-Red, together with test compounds as described in *Material and Methods*. Test compounds were assessed for their ability to modulate the homogeneous time resolved fluorescence resonance energy transfer (HTR-FRET) signal between Lumi4-TB and the red fluorescent acceptor. Vc1.1, ImI (which shares the first 8 amino acids of Vc1.1) (Johnson et al., 1995), AuIB, and the closely related AuIA (Luo et al., 1998) were examined (see Table 2 for peptide sequences). No displacement CGP54626-Red by any of the conotoxins was observed (Figure 5A), indicating that the toxins do not bind to the orthosteric GABA_B_ binding site. We next assessed for agonist activity of conotoxins at G-protein-coupled GABA_B_Rs using a bioluminescence resonance energy transfer (BRET) assay. Agonist binding to GABA_B_R activates heterotrimeric G proteins. HEK cells expressing GABA_B_R coupled to BRET chomophore labeled G-proteins (Gαi_1_/αo_A_-Rluc, Gβ2, and Gγ2-Venus) were utilized to monitor interaction between Gα_i_ or Gα_o_ and Gγ2. The association between Gαi/o-Rluc and Gγ2-Venus at basal state produces a high FRET signal. GABA_B_ binding to the GABA_B_R induces G-protein activation and movement of the Gγ2 subunit away from the Gαi/o subunit leading to a decrease in BRET. GABA robustly decreased BRET, consistent with agonist activity whereas the conotoxins had no effect (Figure 5B).

**Table 2:** Amino acid sequence of selected α-conotoxins.

**Conotoxin**	**Sequence**
RgIA4	GCCTDPRC^*^‡QCY
Vc1.1	GCCSDPRCNYDHPEIC#
AuIA	GCCSYPPCFATNSDYC#
AuIB	GCCSYPPCFATNPD-C#
AnIC	GGCCSHPACFASNPDYC#
ImI	GCCSDPRCAWRC#

* = citrulline

‡ = 3-Iodo-Tyrosine

# = C-terminal amidation

*Peptide sequences of α-conotoxins are shown. For conotoxin name, the two letter code representing Conus species are as follows: Rg, regius; Vc, victor; Au, aulicus; An, anemone; and Im, imperialis*.

## Discussion

α-Conotoxins are small, disulfide rich peptides found in the venom of cone snails. These peptides are typically 12–20 amino acids in length. Some have high subtype selectivity for mammalian nAChRs enabling the dissection of functional roles of these subtypes (Azam and McIntosh, 2009; Lebbe et al., 2014; Dutertre et al., 2017). α-Conotoxin RgIA potently blocks α9α10 vs. other nAChRs subtypes in rat. However, an amino acid difference in the (–) face of the α9 subunit in human vs. rat renders low potency for the α9α10 nAChR (Azam and McIntosh, 2012; Azam et al., 2015). To overcome this species difference, we developed a peptide analog of RgIA, known as RgIA4, wherein 5 of the 13 amino acids were modified. These modifications led to high potency and selectivity for the human α9α10 nAChR (Romero et al., 2017). In this report we have assessed the potency and selectivity of RgIA4 for mouse nAChRs. To do so, we cloned mouse α9 and α10 subunits and placed each of these in a high expression vector suitable for expression in *Xenopus* oocytes.

Application of acetylcholine on mouse α9α10 nAChRs expressed in oocytes induced robust currents. Choline, although sometimes referred to as an α7 selective agonist, also potently activated α9α10 nAChRs consistent with what has been reported for rat and human α9α10 nAChRs (Elgoyhen et al., 1994, 2001; Verbitsky et al., 2000; Sgard et al., 2002). The α9α10 nAChR, as expressed in oocytes and natively expressed in cochlear hair cells, is unique among nAChR subtypes in that it is not activated by nicotine (Elgoyhen et al., 2001). Likewise, nicotine did not activate *Xenopus* oocyte-expressed mouse α9α10 nAChRs. Recently, however, nicotine has been shown to act as an agonist of α9^*^ nAChRs in immune cell monocytes (Hecker et al., 2015; Richter et al., 2016; Backhaus et al., 2017). These nAChRs do not appear to function as canonical ion channels (no ACh- or nicotine-induced ion currents are observed in patch clamp experiments). In these particular cells, agonist stimulation results in alteration of cytokine release. The full subunit composition and structure of immune cell α9^*^ nAChRs is unknown. In some instances, these immune cell nAChRs may be composed of a combination of α7, α9, and α10 subunits (Hecker et al., 2015; Backhaus et al., 2017).

The α7, α9, and α10 subunits are closely related subunits and are the only nAChRs thus far demonstrated to function without a β subunit partner. Interestingly all three subunits may provide the principal or (+) face of the agonist binding interface (Boffi et al., 2017). The parent peptide, RgIA binds to the α10/α9 subunit interface (Azam and McIntosh, 2012; Azam et al., 2015). RgIA4 potently blocked mouse α9α10 nAChRs and the block was selective for this subtype. Block was only slowly reversed upon peptide washout, a characteristic that may allow for synthesis of useful fluorescently or radioactively tagged derivatives. The next most potent block by RgIA4 was that of α7 homomers, but here the IC_50_ was 3,700-fold lower than that of α9α10 and recovery occurred following 2 min of peptide washout. Thus, as for rat and human, RgIA4 represents a novel probe for dissecting function of mouse α9α10 nAChRs vs. α7 nAChRs. Conversely, α-conotoxin [V11L;V16D]ArIB (Whiteaker et al., 2007) potently blocks mouse α7 nAChRs but not α9α10 nAChRs indicating that RgIA4 and [V11L;V16D]ArIB may be used as complimentary reagents.

Increasing lifespan is associated with rising cancer incidence. Cancer chemotherapy is increasingly lifesaving, but associated with significant side effect burden. Commonly used chemotherapeutic agents, including platinum-based drugs, taxanes and thalidomide analogs, are neurotoxic. Neuropathy is one of the most common chemotherapy induced side effects (Banach et al., 2017). The severity of acute peripheral neuropathy is a significant risk factor for severe chronic neuropathy (Hershman et al., 2014). Oxaliplatin is used as a first-line treatment for colorectal cancer. However, oxaliplatin-induced neuropathy can limit both the dose and duration of therapy (Avan et al., 2015). RgIA4 was recently shown to prevent the development of oxaliplatin-induced cold allodynia, an effect which lasted up to 72 h after last injection of peptide (Romero et al., 2017). Here we further examined the potential duration of the analgesic effect of RgIA4. Co-administration of RgIA4 with oxaliplatin prevented the development of cold allodynia. After cessation of oxaliplatin, cold allodynia continued in control mice, but was not observed at any measured time point in mice treated with RgIA4. Thus, RgIA4 treatment prevented the development of oxaliplatin-induced cold allodynia and had prolonged efficacy (total experiment length was 6 weeks). These prolonged effects suggests that similar agents might be administered, along with oxaliplatin, during cancer chemotherapy as a method to provide potentially disease modifying effects with respect to development of neuropathy.

Several conotoxins or derivatives have now been reported to have analgesic effects. Some of these toxins block α9α10 nAChRs as expressed in oocytes, yet others, such as α-conotoxin AuIB do not (Klimis et al., 2011). These findings have called into question whether the analgesic effects involve an α9α10 nAChR dependent pathway. In addition, several reports have indicated that conotoxins, including Vc1.1, analogs of Vc1.1, RgIA and AuIB block N-type calcium channels in DRG neurons, an effect that is prevented by GABA_B_R antagonists (Callaghan et al., 2008; Callaghan and Adams, 2010; Klimis et al., 2011; Cuny et al., 2012; van Lierop et al., 2013; Berecki et al., 2014; Huynh et al., 2015; Castro et al., 2016). This has led to an alternative hypothesis that these conotoxins exert their analgesic effect by stimulating G-protein coupled GABA_B_R (Adams et al., 2012; Adams and Berecki, 2013; Sadeghi et al., 2017). In support of this, post-translationally modified analogs of Vc1.1 that have α9α10 activity but lack GABA_B_R activity also lack analgesic activity (Nevin et al., 2007). However, several laboratories have failed to observe conotoxin-evoked GABA_B_R responses; these assays have utilized DRG neurons, spinal cord slices and *Xenopus* oocyte-expressed GABA_B_R (Xiao et al., 2009; Napier et al., 2012; Huynh et al., 2015; Wright et al., 2015).

RgIA4 was previously shown to not stimulate GABA_B_R as expressed in DRG or immortalized cell lines (Romero et al., 2017). To further evaluate the potential structure function relationship of other conotoxins on GABA_B_R we used a FRET-based binding assay and functional BRET assay. Peptides analyzed included Vc1.1, AuIB, and three other conotoxins with similar amino acid sequences (Table 2). None of these conotoxins prevented the binding of the competitive orthosteric antagonist CGP54626-Red. This is consistent with previous binding studies performed in which Vc1.1 did not compete for binding with [3H]CGP54626 in HEK293T cells (McIntosh et al., 2009) or in DRG neurons (Adams and Berecki, 2013). The latter studies led to the proposal that Vc1.1 acts at an allosteric site to exert its agonist effect (Adams et al., 2012; Adams and Berecki, 2013). However, in the current study, neither Vc1.1 nor any of the examined conotoxins activated the GABA_B_R as measured with a G-protein-based BRET assay. This is surprising given that Vc1.1 activation of GABA_B_Rs was previously reported to be dependent on the G-protein signaling cascade; block of N-type calcium channels was eliminated by pertussis toxin, intracellular GDPβS, or an inhibitor of pp60c-src tyrosine kinase (Callaghan et al., 2008).

We are unsure how to reconcile the GABA_B_R mediated effects reported in multiple compelling studies using DRG neurons (Callaghan et al., 2008; Callaghan and Adams, 2010; Klimis et al., 2011; Cuny et al., 2012; van Lierop et al., 2013; Berecki et al., 2014; Huynh et al., 2015; Castro et al., 2016), (but see Wright et al., 2015 that did not replicate principle findings of these studies) and the lack of effects seen in other assays (McIntosh et al., 2009; Napier et al., 2012), now including the GABA_B_R BRET assay. There is known interplay between nicotinic and GABAergic systems. CNS nAChRs modulate GABA release (Radcliffe et al., 1999). In addition, GABA_B_ autoreceptors inhibit ACh-evoked GABA release (McClure-Begley et al., 2014). However, Vc1.1 and RgIA were shown to block N-type calcium channels in cultured neurons from α9 KO mice indicating that block of α9 nAChRs could not account for GABA_B_R effects observed in that preparation (Callaghan and Adams, 2010). Cross-regulation of cholinergic and GABAergic systems may also be indirect. For example, microRNAs can repress multiple targets; miRNA-608 targets both acetylcholinesterase and the Rho GTPase CDC42 that is involved in GABAA synapse formation (Hanin et al., 2014). In the present study, the primary GABA_B_ signaling complex (the GABA_B_R and G-proteins α, β, and γ subunits) was expressed in HEK cells. It is possible that in DRG neurons other additional and unknown protein partners may be involved and necessary for conotoxins to act as an agonist. Regardless, the present findings become an interesting piece of a puzzle that must be reconciled with the GABA_B_R hypothesis.

Acute pain is protective to the animal; yet pain can extend for weeks or years, well after healing of the initial injury, and well beyond pain's adaptive utility. Another intriguing finding that must be explained is how these α-conotoxins produce their long lasting effects. RgIA4, a peptide, is rapidly cleared from plasma with a half-life of < 20 min (Mercado et al., 2016). Plasma levels are undetectable at 4 h. Despite the slow reversal of RgIA4 action, block (or stimulation) of any receptor would not be expected to be present for weeks after last compound administration. How then to explain these long lasting effects? The answer is unknown at the present time, but one possible clue comes from examination of the histopathological effects of Vc1.1 and RgIA administration. Nerve injury produces an influx of T-cells and macrophages into the area of nerve injury (Austin et al., 2012; Ji et al., 2016; Lees et al., 2017). Some of these immune cell responses may help to heal damaged tissue while others may lead to further pathology. Alterations in microglia are also implicated in neuropathic pain states; RgIA was also shown to alter levels of CNS microglia (Di Cesare Mannelli et al., 2014). The interplay between neurons and immune cells are increasingly recognized as important in the pathophysiology of neuropathic pain (Austin et al., 2012; Grace et al., 2014; Ji et al., 2016; Peng et al., 2016; Lees et al., 2017). It is possible that the α-conotoxins modulate subpopulations of immune cells in a fashion that favorably alters the development of a pain state. There is precedent for attenuation of neuroimmune signaling to produce long lasting reversal of neuropathic pain. A single injection of adenosine 2A receptor agonists attenuated chronic constriction injury-induced upregulation of spinal cord microglia and astrocytes and reversed mechanical and thermal hyperalgesia, effects that lasted at least 4 weeks (Loram et al., 2009, 2013). Functional α9α10 nAChRs have recently been reported in monocytes where they influence P2X receptor release of IL-1β (Hecker et al., 2015; Richter et al., 2016; Backhaus et al., 2017). Transcripts for α9 and α10 subunits have been reported in other immune cell subtypes (Hao et al., 2011). The functions of α9α10 receptors in these cells, and those that are affected by nerve injury are unknown.

Analgesic effects of nAChR antagonists are not limited to the α-conotoxins. Small molecule antatonists of α9α10 nAChRs have been shown to have analgesic effects in chronic constriction injury and chemotherapy (vincristine)- induced neuropathy (Holtman et al., 2011; Zheng et al., 2011; Wala et al., 2012) (see Hone et al., 2017 for review). Another α9α10 nAChR antagonist conotoxin, GeXIVA (Luo et al., 2015) structurally unrelated to the α-conotoxins was also shown to produce long lasting analgesic effects (Li et al., 2016). These findings together with those of the present report further implicate α9α10 nAChRs in the treatment of neuropathic pain.

## Author contributions

SC, AH, IR, JK, and GU all performed the experiments. SC, AH, IR, JK, JP, GU, DS, EG, and JM all participated in experimental design and the preparing and writing of the manuscript.

### Conflict of interest statement

Conotoxins, including some of those referenced in this paper have been patented by the University of Utah with JM listed as an inventor. JM has received funding from Kineta Inc. The other authors declare that the research was conducted in the absence of any commercial or financial relationships that could be construed as a potential conflict of interest.
